# An unanticipated case of congenital high airway obstruction syndrome with type C tracheoesophageal fistula and cerebral ventriculomegaly in a preterm infant – is a small stomach a remote clue?

**DOI:** 10.1515/crpm-2025-0016

**Published:** 2026-08-24

**Authors:** Anitha Irakam, Poonam Nayak, Amrita Sunkad, Bellipady Rai

**Affiliations:** Division of Neonatology, Department of Pediatrics, Children’s Hospital of New Jersey, Newark, NJ, USA

**Keywords:** congenital high airway obstruction syndrome, esophageal atresia, tracheoesophageal fistula, small stomach, cerebral ventriculomegaly, bilateral hypoplastic lungs

## Abstract

**Objectives:**

To detail the clinical presentation, diagnostic challenges and autopsy findings of a neonate with unanticipated Congenital High Airway Obstruction Syndrome (CHAOS) and esophageal atresia (EA) with tracheo-esophageal fistula (TEF), while noting the small stomach as an observational prenatal finding in the absence of polyhydramnios that may prompt evaluation of the upper airway.

**Case presentation:**

Thirty one weeks male neonate was born by vaginal delivery with severe respiratory distress following preterm prolonged rupture of membranes (PPROM) and placental abruption presenting with profound hypotonia and low Apgar scores. Antenatal imaging at 20 and 22 weeks had confirmed a small stomach and bilateral cerebral ventriculomegaly but consistently with normal amniotic fluid. Attempts at endotracheal intubation failed as the ETT could not be advanced past the vocal cords. During positive pressure ventilation (PPV), air was seen puffing in the neck, raising suspicion for upper airway obstruction. Emergency tracheostomy failed to achieve ventilation. Resuscitation was stopped about 90 min of life. Autopsy report showed congenital laryngeal/tracheal atresia at the level of the cricoid cartilage, proximal esophageal atresia with Type C TEF, hypoplastic lungs and a small stomach.

**Conclusions:**

This case suggests that small stomach may be an observational prenatal finding for associated esophageal obstruction, prompting evaluation of the upper airway, especially in the setting of normal amniotic fluid. A high degree of suspicion for CHAOS should guide management in neonates presenting with severe respiratory distress and failed intubation. If difficult intubation is anticipated, ENT team availability will help for immediate tracheostomy.

## Introduction

Laryngeal/tracheal atresia or Congenital High Airway Obstruction Syndrome (CHAOS), esophageal atresia (EA) with tracheo-esophageal fistula (TEF) is a rare congenital anomaly resulting from absent recanalization of the upper airway and incomplete separation of the trachea and esophagus during embryonic development. The incidence of tracheal atresia is approximately 1 in 50,000 births [1]. Incidence of TEF is 1:2500 [2]. But the true incidence of CHAOS with or without TEF remains unknown [3]. Despite advancements in antenatal imaging, airway obstruction is often diagnosed postnatally following a critical presentation at birth. The classic ultrasound findings are dilated airways below the level of obstruction, hyperexpanded and hyperechoic lungs and flattened diaphragm, but CHAOS remains a frequently missed diagnosis [3]. CHAOS typically presents as a neonatal emergency with symptoms such as aphonia, severe respiratory distress, cyanosis, and hypotonia at birth. Postnatal diagnostic signs include failed attempts at intubation with no evidence of air entry during PPV. A visible air puffing in the neck strongly suggests atresia of both the respiratory tract and gastrointestinal tract. This case report details the clinical presentation, diagnostic challenges, and autopsy findings of a neonate born at 31 weeks of gestation with unanticipated CHAOS and EA with TEF without signs of polyhydramnios. An early antenatal observation of a small stomach may offer an additional clue in the absence of polyhydramnios and may warrant fetal radiology consultation, while recognizing that it remains an observational finding rather than a definitive diagnostic indicator (Table 1).

**Table 1:** Comparative analysis of reported CHAOS + TEF cases.

#	Title	Author	Year	Key features	Outcomes reported	How this differs from our case
1	Congenital absence of trachea	Payne WA	1900	Isolated tracheal absence	Fatal shortly after birth	Our case involves combined laryngeal, tracheal, and esophageal anomalies
2	Congenital tracheal obstruction with esophageal atresia	Ashley D	1972	Tracheal + esophageal obstruction	Poor survival	No laryngeal involvement in this case
3	Tracheal atresia, proximal EA and distal TEF	Sankaran et al.	1983	TA + EA + TEF; polyhydramnios	Neonatal mortality reported	Our case lacked polyhydramnios
4	Long-term survival in laryngeal atresia	Okada	1998	Laryngeal, intestinal, urethral anomalies	Long-term survival achieved	Additional systemic anomalies not present in our case
5	Tracheal agenesis with bronchoesophageal fistulas	Demircan et al.	2008	TA + EA + BEF	Poor prognosis	No TEF; differs anatomically
6	CHAOS syndrome	Mudaliar et al.	2017	CHAOS with hyperechoic lungs	Typically, fatal without intervention	Our case had small (hypoplastic) lungs
7	CHAOS + EA + TEF + duodenal atresia	Kanamori et al.	2017	Multiple GI anomalies	Poor outcome	Additional duodenal atresia not seen in our case
8	CHAOS survival with laryngeal atresia	Heriseanu et al.	2023	Isolated laryngeal atresia	Survival with intervention	Our case involves multi-level airway involvement
9	Laryngeal atresia with H-type TEF	Soni et al.	2024	Laryngeal atresia + H-type TEF	Managed with airway intervention	Our case had type C TEF
10	Tracheal agenesis with TEF	Wu et al.	2024	TA + TEF without EA	Poor survival overall	Our case includes esophageal atresia

## Case presentation

**Figure 1:**
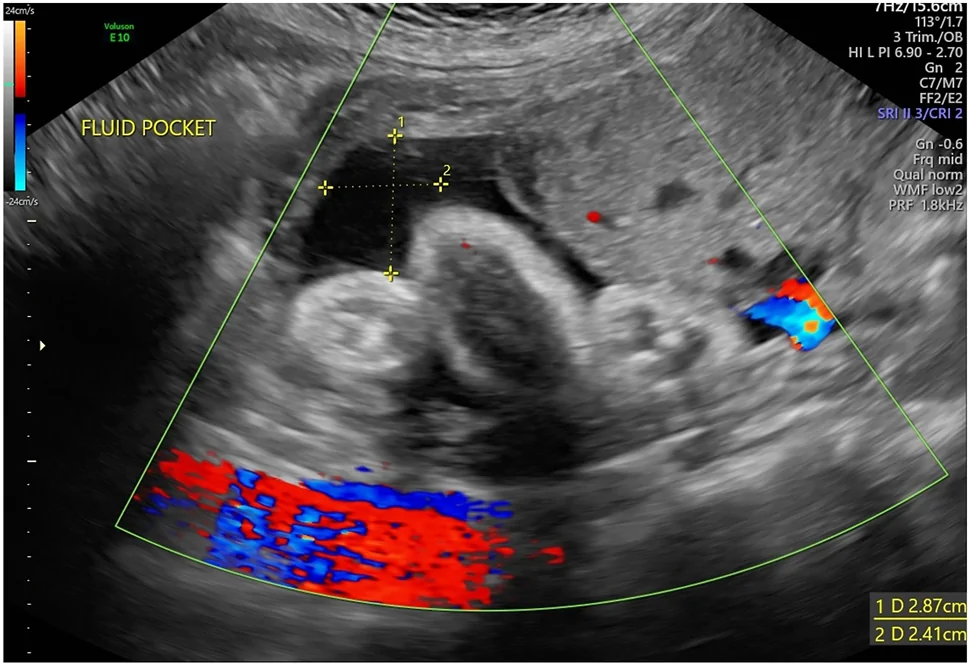
Ultrasound showing normal amniotic fluid.

**Figure 2:**
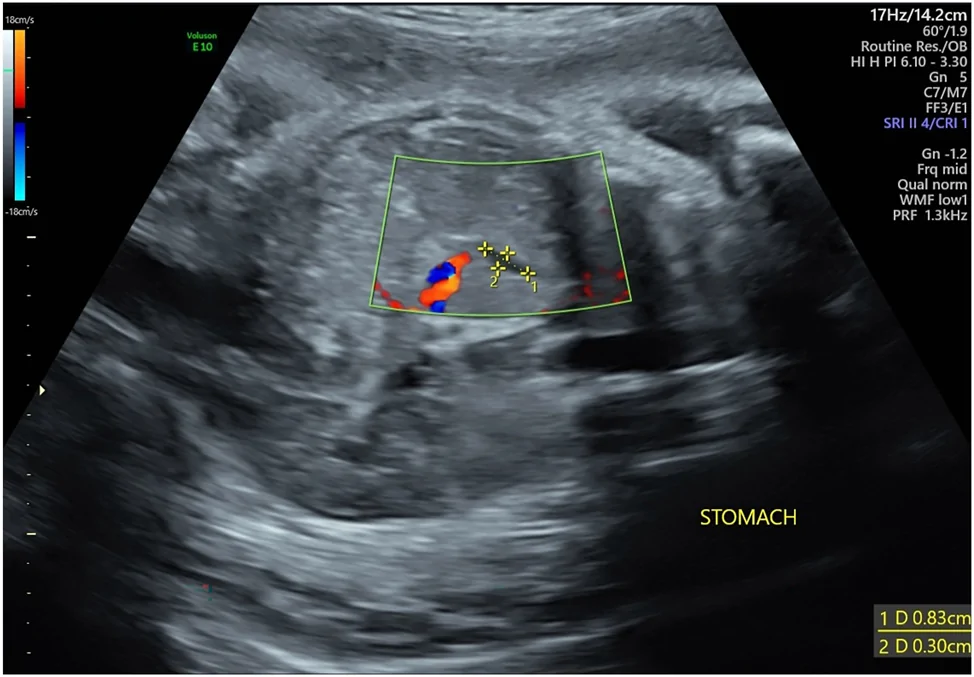
Ultrasound showing small stomach.

**Figure 3:**
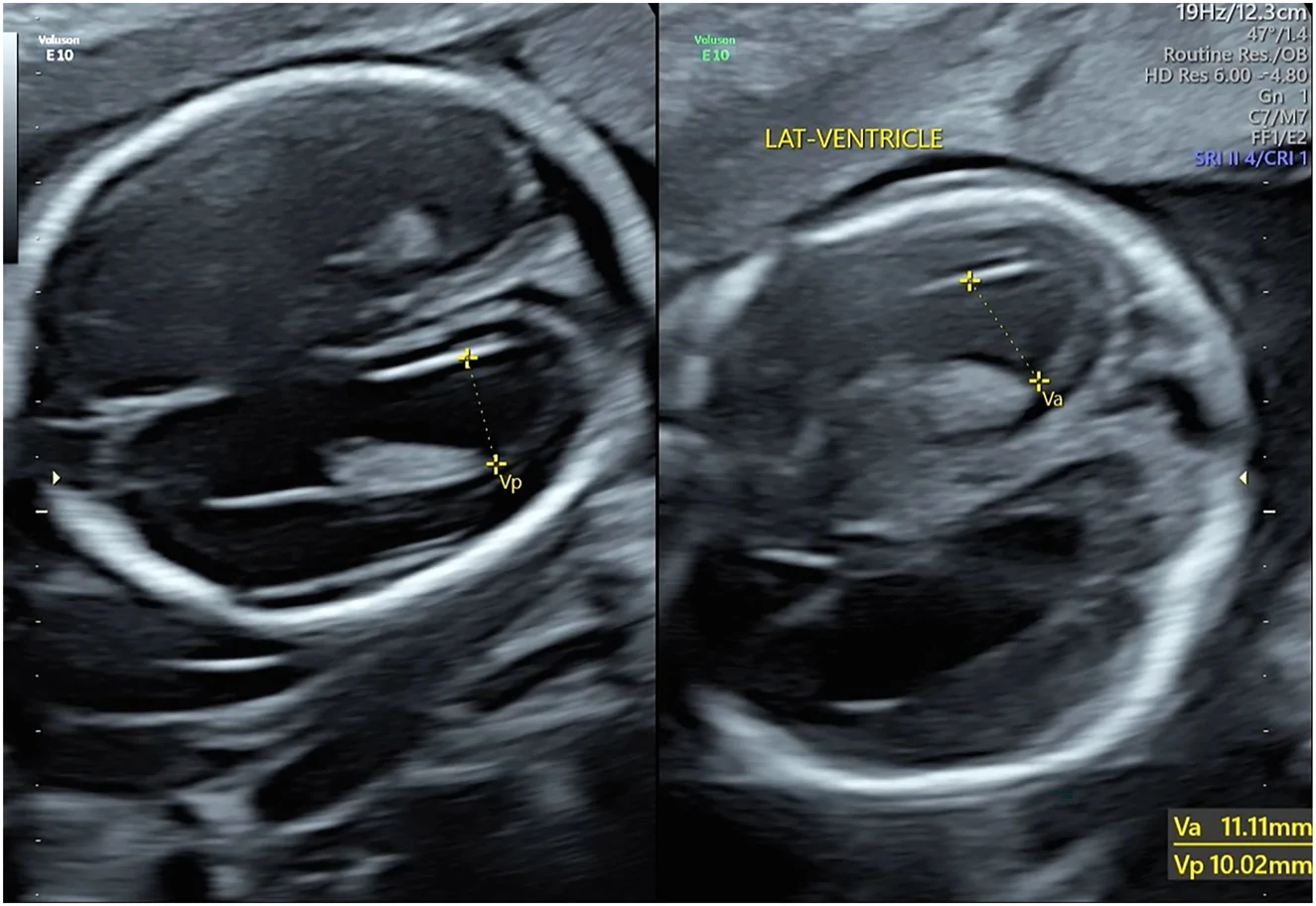
Ultrasound showing cerebral ventriculomegaly.

**Figure 4:**
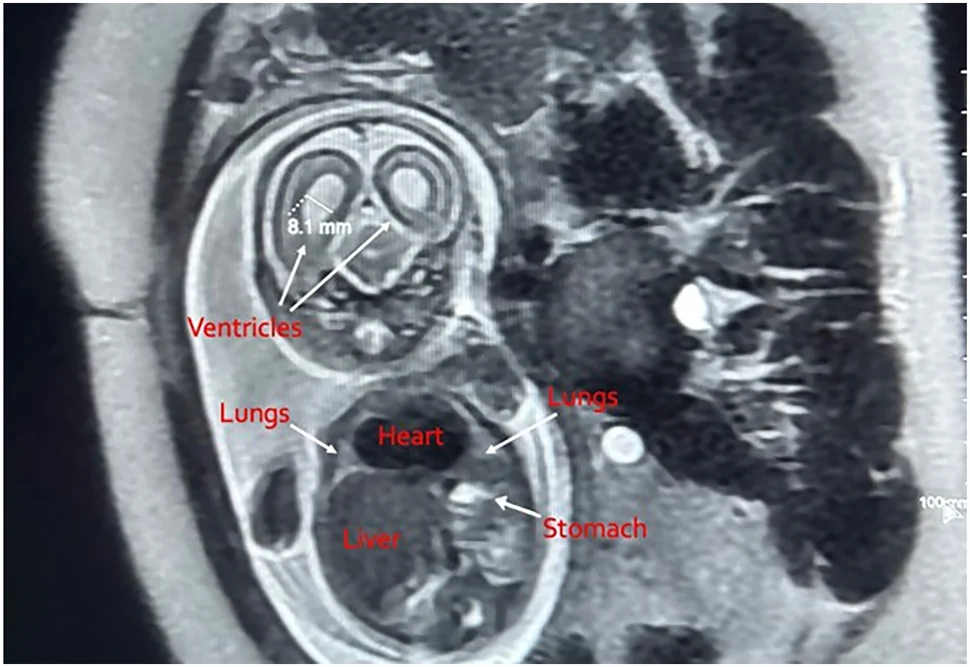
Fetal MRI showing cerebral ventriculomegaly, small stomach, normal amniotic fluid volume.

**Figure 5:**
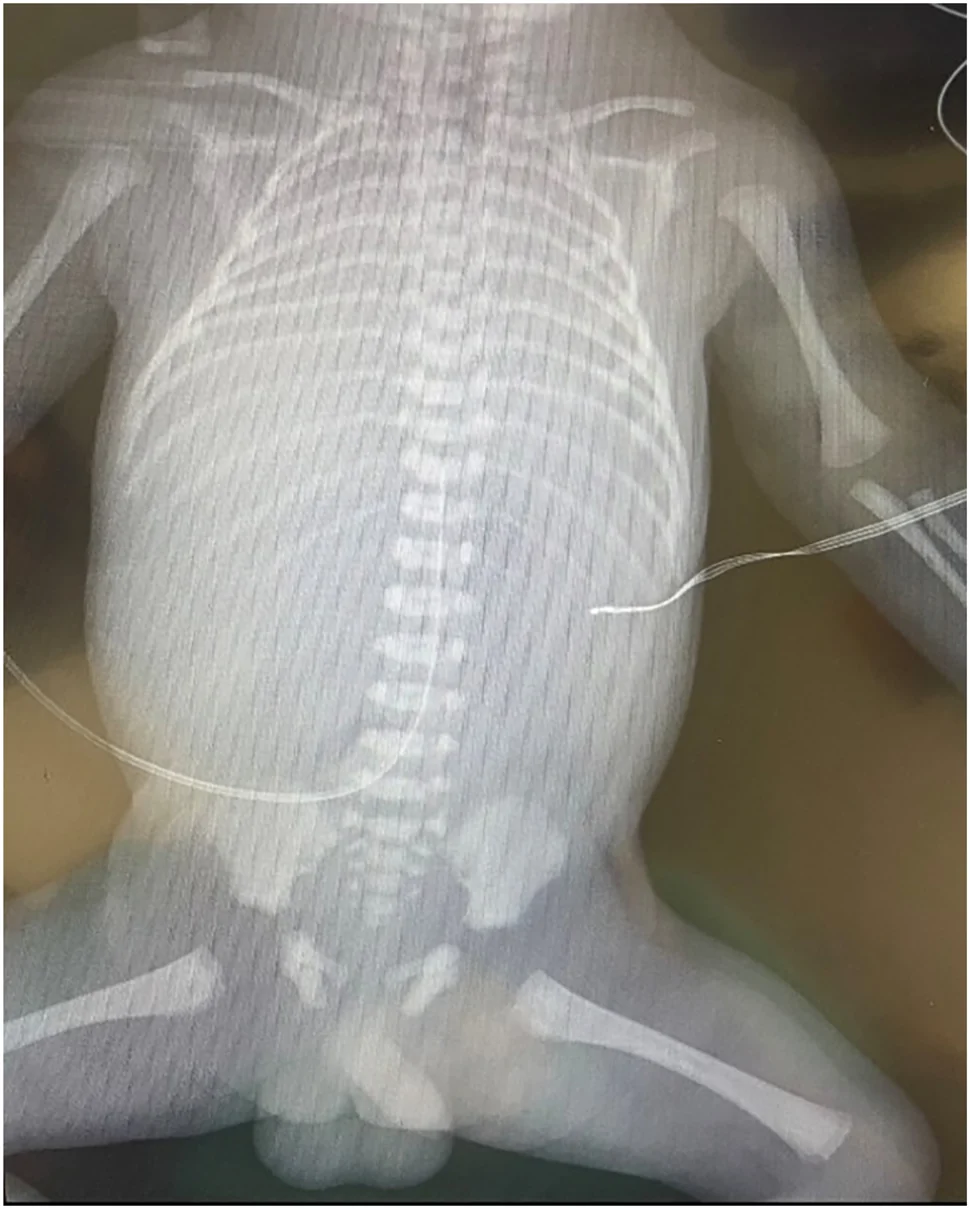
X-ray showing complete opacification of lungs and abdomen, small chest and dilated esophagus.

A male neonate was born preterm at 31 3/7 weeks of gestation via spontaneous vaginal delivery complicated by PPROM and placental abruption. Fetal ultrasound at 20 weeks was remarkable for normal amniotic fluid (Figure 1), a small stomach (Figure 2), bilateral cerebral ventriculomegaly with absent cavum septum pellucidum (Figure 3), prompting a fetal MRI at 22 weeks. While limited due to fetal movement during imaging, MRI confirmed bilateral cerebral ventriculomegaly and a small stomach with normal amniotic fluid (Figure 4). After a few weeks of missed appointments, the mother was admitted at 28 weeks with PPROM where she received prophylactic antibiotics and a full course of antenatal steroids in anticipation of preterm delivery. 18 days later at 31 3/7 weeks of gestation, complicated by severe vaginal bleeding, she delivered a neonate who presented with cyanosis, pallor, profound hypotonia, and absence of cry with no spontaneous respiratory effort. Immediate warming, drying, stimulation, and suctioning was done. The heart rate remained below 100 bpm with low oxygen saturations. No spontaneous activity noted. PPV was initiated immediately due to absent respiratory effort and bradycardia. There was only a brief improvement in saturations and heart rate, so the baby was presumed intubated at 2 min of life with some improvement in heart rate and saturations on 100 % oxygen at 5 min. Placental abruption was reported at this time. The patient continued to have desaturations and heart rate below 60 bpm requiring chest compressions. An emergency umbilical venous line (UVL) was placed with multiple rounds of epinephrine and normal saline bolus given for possible hypovolemia secondary to placental abruption with no response. Multiple reintubation attempts failed, as the ETT could not be advanced beyond the normal appearing vocal cords. The LMA attempt was also unsuccessful. During PPV with bag and mask, air was seen puffing in the neck while the chest exhibited no movement raising suspicion of severe upper airway obstruction. An attempt to pass an orogastric tube also failed. The baby’s heart rate remained persistently less than 100 bpm with low saturations prompting transfer to the NICU for emergency ENT evaluation and tracheostomy. Apgar scores were 1, 2, 2 at 1, 5 and 10 min of life respectively. Physical exam of the baby revealed a floppy baby, with no dysmorphic features and a two-vessel cord. X-ray revealed complete opacification of lungs and abdomen with a dilated upper esophagus and a small chest (Figure 5). An emergency bedside tracheostomy was performed by the ENT team. Airway management was taken over by ENT team and anesthesiologists. Attempts on intubation were made with different sizes of ETT. Video laryngoscopy guided intubation was eventually performed, demonstrating the tip of ETT barely passing through the vocal cords and effective ventilation could not be achieved. A rigid bronchoscopy was then performed, direct visualization beyond the vocal cords revealed a blind end suggesting possible laryngeal atresia. Emergency tracheostomy was performed at 51 min of life. Copious serous fluid was immediately suctioned from the trachea. Minimal breath sounds were noted with PPV via tracheostomy with no chest rise. Continued efforts were unsuccessful in ventilating the lungs. Despite exhaustive resuscitative measures, the neonate could not be ventilated and was pronounced dead within 2 h of life. The parents consented to an autopsy excluding the brain.

### Autopsy findings

**Figure 6:**
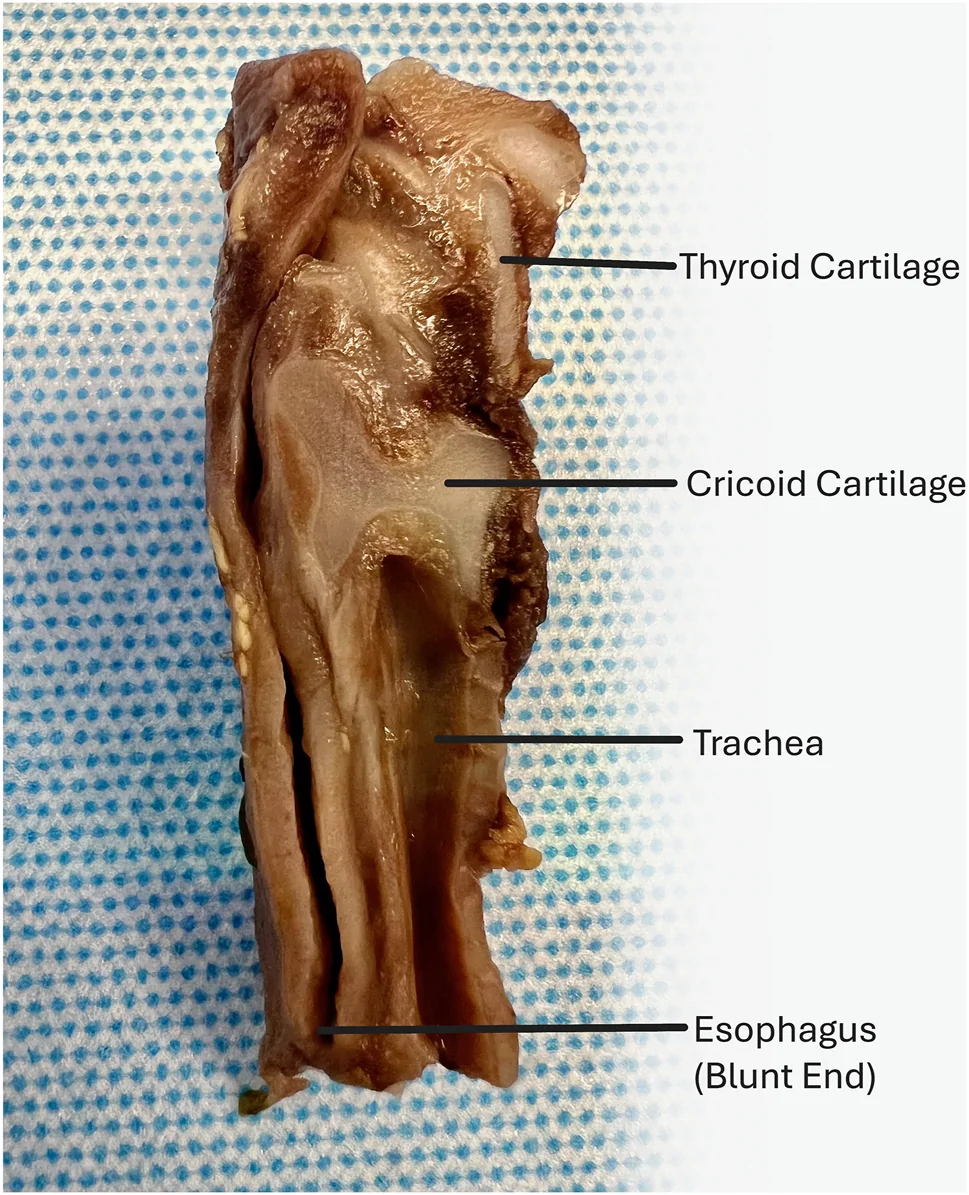
Sagittal autopsy section demonstrating proximal esophageal atresia with a blind pouch with laryngeal/tracheal atresia at the level of cricoid cartilage.

Autopsy revealed a 2.11 kg male neonate with congenital laryngeal/tracheal atresia at the level of the cricoid cartilage, pulmonary hypoplasia, and a two-vessel cord. The proximal portion of the esophagus appeared to end in a blind pouch (Figure 6). Distally, the esophagus appeared to rearise from the trachea and was patent to the stomach consistent with Type C TEF. The stomach appeared small but normally located in the left upper quadrant.

## Discussion

In the embryological period, the formation of a laryngotracheal groove by four weeks is the first indication of the respiratory tract. The larynx develops from the cranial end of this groove while the trachea develops from the middle part of the groove. The laryngotracheal groove deepens to form a diverticulum anterior to the pharynx. The distal portion of this diverticulum will eventually grow into bronchial buds which further develops into lungs. Proximally, the lateral walls of the diverticulum invaginate to form tracheoesophageal folds, which eventually fuse in the midline. This fusion forms a tracheoesophageal septum which separates the primitive airway from the esophagus. The cartilages of the larynx are derived from the fourth and sixth pair of branchial arches in the fifth week of gestation. Arytenoid swellings form at the laryngeal inlet, narrowing the slit like glottis. The glottic epithelium proliferates rapidly and temporarily occludes the lumen. Recanalization of this lumen occurs at the 10th week of embryonic life. Any interruption in the embryological development during recanalization leads to congenital laryngeal and tracheal abnormalities. Any disruption in the formation of the tracheoesophageal septum leads to various types of tracheoesophageal fistula [4]. The most common variety is type C, where the proximal esophagus is dilated and blind ending is about the level of the third or fourth thoracic vertebra, with a distal esophagus which is thinner and narrower attached to the posterior wall of the trachea near the carina [2].

Overview of the triad involving polyhydramnios, gastrointestinal obstruction, and a small fetal stomach indicates a physiological disruption where the fetus cannot swallow or process amniotic fluid due to structural or neurological issues. Congenital High Airway Obstruction Syndrome (CHAOS) triggers this triad through extrinsic structural compression. A complete blockage of the fetal larynx or trachea traps lung fluid, causing the lungs to hyper-expand under high intrathoracic pressure. This expansion physically squeezes the adjacent esophagus, acting as a high GI obstruction. This compression prevents amniotic fluid from reaching the stomach, leaving the gastric walls uninflated and abnormally small on ultrasound. Because the fetus cannot swallow or recycle the fluid while urination remains normal, the unabsorbed fluid accumulates in the uterine cavity, resulting in severe polyhydramnios.

Neonatal resuscitation is performed when a newborn fails to establish effective breathing or circulation at birth. The pathophysiology underlying the need for resuscitation involves complex transitions from intrauterine to extrauterine life. When this transition is disrupted, a cascade of birth asphyxia occurs. Failure to aerate the lungs due to obstruction at any level leads to prolonged hypoxia and myocardial dysfunction. Positive Pressure Ventilation is the most critical step, as improving oxygenation typically resolves the secondary bradycardia and potentially cardiac arrest.

CHAOS is a rare congenital anomaly caused by laryngeal or tracheal atresia, tracheal stenosis, obstructing laryngeal cysts, obstructing tumors of the oropharynx and the cervical region, or compression from a double aortic arch. Immediate postnatal management is challenging with efforts to secure the airway. CHAOS should be an important diagnosis to be considered in neonates presenting with severe respiratory distress and no audible cry at birth. Endotracheal intubation cannot be accomplished because it is impossible to advance the tube beyond the vocal cords. Immediate tracheostomy can be lifesaving; however, it necessitates a patent distal tracheal lumen to be effective. If CHAOS has been antenatally identified EXIT procedure should be considered which increases the rate of survival for these patients [5]. LMA may be lifesaving if there is difficulty with intubation but ability to ventilate raises the possibility of a H type fistula with CHAOS [6]. If tracheal intubation attempts are unsuccessful, esophageal intubation could be a vital component of neonatal resuscitation to increase the chance of successfully transitioning to surgical reconstruction [7].

Prenatal ultrasound and fetal MRI are the standards for detection of CHAOS which includes laryngeal/tracheal atresia. The key findings on a prenatal ultrasound include enlarged, hyper echogenic lungs, a dilated tracheobronchial tree, a flattened or an inverted diaphragm and ascites all indicating a blockage in the upper airway of the fetus [5]. Further confirmation may be achieved with a fetal MRI to pinpoint the location of obstruction. Few such cases have been reported and some who included survival had a history of polyhydramnios and were prenatally diagnosed [8]. In our case, absence of polyhydramnios starting with ultrasound at 20 weeks, fetal MRI at 22 weeks and subsequent antenatal ultrasounds as late as 31 weeks with no evidence of hyperechogenic lungs due to TEF prevented suspicion of CHAOS. Early presence of a small stomach could have been a clue of gastrointestinal tract obstruction combined with airway obstruction. Though small stomach could also be due to physiological, structural, anatomical, or chromosomal defects, an absent stomach or a small stomach after 18 weeks gestation is associated with a guarded prognosis [9], [10], [11], [12]. The first description of tracheal atresia was described by William Payne in 1900 [13] where he describes a case with isolated tracheal atresia with no tracheoesophageal connections. Then the first case of both tracheal atresia and esophageal atresia with TEF was described by David Ashley in 1972 [14]. Successful surgical repair of TEF was not achieved until 1941, when Cameron Haight performed the inaugural procedure with tracheostomy as the mainstay of management [15]. If the trachea is palpable below the atretic area, immediate tracheostomy may be lifesaving [16]. There are recent advances in surgical techniques for management of TEF. Different reconstructive procedures using either the esophagus or synthetic material have been attempted to create an airway lumen [5], 17]. Given the variable and risk of severe presentation, early detection can be a valuable tool to ensure better prognosis. A high degree of suspicion in neonates presenting with severe respiratory distress could help guide the management. Emergency tracheotomy/tracheostomy can also be considered as an alternate airway management during neonatal resuscitation if ETT and LMA fails to achieve ventilation. In our case, findings on prenatal ultrasound failed to show any signs of upper airway or gastrointestinal obstruction except for a small stomach. There are no prior cases of CHAOS reported that are known to be associated with small stomach.

## Conclusions

This case suggests that small stomach may be an observational prenatal finding for associated esophageal obstruction, prompt evaluation of the upper airway, especially in the setting of normal amniotic fluid. A high degree of suspicion for CHAOS should guide management in neonates presenting with severe respiratory distress and failed intubation. When difficulty with intubation is anticipated, early consultation with otolaryngology should be considered so that a team capable of performing a surgical airway is immediately available if required. While the small stomach is only an observational finding, it may offer insight for future cases and guide clinical consideration in similar scenarios.
